# PKC-β modulates Ca^2+^ mobilization through Stim1 phosphorylation

**DOI:** 10.1007/s13258-022-01230-3

**Published:** 2022-03-07

**Authors:** Hye-Jin Song, In-Sook Jeon, Seung Ryul Kim, Kwan Sik Park, Jae-Won Soh, Kwang Youl Lee, Jae-Cheon Shin, Hak-Kyo Lee, Joong-Kook Choi

**Affiliations:** 1 Division of Biochemistry, College of Medicine, Chungbuk National University, Ch’ongju, 28644 Korea; 2 Biomedical Research Center for Signal Transduction Networks, Department of Chemistry, Inha University, Incheon, 402-751 Korea; 3 College of Pharmacy, Chonnam National University, Gwangju, 500-757 Korea; 4 Pohang Center for Evaluation of Biomaterials, 394, Jigok-ro, Nam-gu, Pohang, Gyeongbuk Korea; 5 Department of Animal Biotechnology, Chonbuk National University, Chonju, 54896 Jeollabuk-do Korea

**Keywords:** PKC, Stim1, Store-operated calcium entry, Calcium mobilization, Stim1 phosphorylation

## Abstract

**Background:**

Calcium ions play a pivotal role in cell proliferation, differentiation, and migration. Under basal conditions, the calcium level is tightly regulated; however, cellular activation by growth factors increase the ion level through calcium pumps in the plasma membrane and endoplasmic reticulum for calcium signaling. Orai1 is a major calcium channel in the cell membrane of non-excitable cells, and its activity depends on the stromal interaction molecule 1 (Stim1). Several groups reported that the store-operated calcium entry (SOCE) can be modulated through phosphorylation of Stim1 by protein kinases such as extracellular signal-regulated kinase (ERK), protein kinase A (PKA), and p21-activated kinase (PAK). PKC is a protein kinase that is activated by calcium and diacylglycerol (DAG), but it remains unclear what role activated PKC plays in controlling the intracellular calcium pool.

**Objectives:**

Here, we investigated whether PKC-β controls intracellular calcium dynamics through Stim1.

**Methods:**

Several biochemical methods such as immune-precipitation, site directed mutagenesis, in vitro kinase assay were employed to investigate PKC interaction with and phosphorylation of Stim1. Intracellular calcium mobilization, via Stim1 mediated SOCE channel, were studied using in the presence of PKC activator or inhibitor under a confocal microscope.

**Results:**

Our data demonstrate that PKC interacts with and phosphorylates Stim1 in vitro. phosphorylation of Stim1 at its C-terminal end appears to be important in the regulation of SOCE activity in HEK293 and HeLa cells. Additionally, transient intracellular calcium mobilization assays demonstrate that the SOCE activity was inhibited by PKC activators or activated by PKC inhibitors.

**Conclusion:**

In sum, our data suggest a repressive role of PKC in regulating calcium entry through SOCE.

**Supplementary Information:**

The online version contains supplementary material available at 10.1007/s13258-022-01230-3.

## Introduction

The Ca^2+^ ions are essential regulator of many normal cellular processes, including muscle contraction, gene transcription, and cell migration (Bong and Monteith 2018; Carafoli 2002; Clapham 2007; Kim et al. 2014; Wei et al. 2012). However, dysregulated calcium signaling appears to be involved in cancer cell proliferation, invasion, and sensitivity to cell death (Jeon et al. 2018, Monteith et al. 2017, Roberts et al. 2019; Tsai et al. 2014).

Intracellular Ca^2+^ levels are tightly controlled in non-excitable cells by ligand-gated Ca^2+^ channels in the plasma membrane (PM) and endoplasmic reticulum (ER). The activation of store-operated calcium entry (SOCE) can be initiated by various ligands, such as growth factors, neurotransmitters or Thapsigargin (TG), and is propagated by phospholipase C (PLC) through the production of inositol triphosphate (IP3) and IP3 binding to ER-resident calcium channels, resulting in the release of Ca^2+^ from the ER store (Liou et al. 2005; Prakriya et al. 2015; Roos et al. 2005). During this calcium depletion process, the N-terminal domain of Stim1 accordingly dissociates from intra ER calcium ions and undergoes conformational changes to promote self-oligomerization, after which it localizes to the PM for interaction with the calcium channel Orai1. The fine-tuning of SOCE and concomitant fluctuation in intracellular Ca^2+^ concentration may allow cells to control calcium-regulated signal pathways to cope with diverse environmental circumstances and facilitate cell migration (Casas-Rua et al. 2015; Luik et al. 2008; Yang et al. 2013).

Many protein kinases, including ERK, PKA, PAK1 and CamKII (Jeon et al. 2018; Pozo-Guisado et al. 2010; Thompson et al. 2015; Li et al. 2018), reportedly phosphorylate Stim1 to increase calcium entry through SOCE. On the other hand, phosphorylation of Stim1 at ERK/CDK sites is reported to be dispensable for cell migration and ER partitioning in mitosis (Hammad et al. 2021). Intracellular ATP depletion by several chemicals including oligomycin, deoxy-d-glucose or ionomycin also increases SOCE conceivably via Stim1 oligomerization (Chvanov et al. 2008; Morgan and De Bondt 1994). On the other hand, AMP-activated protein kinase and Ubiquilin 1 were recently identified to inhibit SOCE by Stim1 phosphorylation and Orai1 degradation, respectively (Lee et al. 2013; Nelson et al. 2019).

Protein kinase C (PKC) is a family of serine/threonine kinases that regulate both positive and negative signaling pathways involved in the immune response, platelet activation, and migration of cancer cells (Harper et al. 2010; Masur et al. 2017; Wen et al. 2018; Xiao et al. 2013). Compared to the above-described protein kinases, classical types of PKC (cPKC), including PKC-α, -β and -γ, appear to be unique because they can be activated by intracellular Ca^2+^ and diacylglycerol (DAG), both of which are downstream effectors of PLC (Huang et al. 1989; Spitaler and Cantrell 2004). However, after PKC activation, it remains unclear whether PKC can modulate Ca^2+^ channel (SOCE) activity as a feedback mechanism. This question led us to study the role of PKC in the regulation of intracellular Ca^2+^ mobilization in two human cell lines (HeLa and HEK293). In an initial screening study for any interaction between PKC and Stim1, we included both classical and novel forms of PKCs, but subsequently, focused on a classical PKC-β since it is widely reported to play diverse roles in many different cellular contexts (Kawakami et al. 2002).

Using immunoprecipitation and Ca^2+^ mobilization and confocal assays, we discovered that (1) PKC-β interacts with and phosphorylates the C-terminal region of Stim1, whereby residue Ser660 represents an important target site, (2) mutation of Ser660 to Ala restores SOCE activity in response to PKC activation, (3) PKC activity is inversely related to SOCE activity, and (4) Stim1 oligomerization induced by ionomycin is affected by either chemical activators or inhibitors of PKC isozymes. Taken together, our data suggest that PKC regulates SOCE through phosphorylation of the C-terminal region and de-oligomerization of Stim1.

## Materials and methods

### Cell lines and reagents

HeLa (human cervical cancer) cells and human embryonic kidney (HEK)293 cells were cultured in Dulbecco’s modified Eagle’s medium (DMEM, Gibco/Waltham, MA, USA) supplemented with 10% fetal bovine serum (FBS, Gibco) and 1 mg/ml penicillin/streptomycin (P/S, Welgene/Gyeongsan-si, Gyeongsangbuk-do, Korea) under 5% CO_2_ at 37 ℃. TG and fluorescent dyes, such as Alexa488 and Alexa594, were purchased for calcium assay from Invitrogen (Carlsbad, CA, USA). The Ca^2+^-sensitive dye Fluo-4 was provided by Thermo Fischer Sci (Waltham, MA, USA). Flip-in T-REx™ 293 cell line was purchased from Invitrogen. Hygromycin B and Ionomycin were obtained from Sigma (St. Louis, MS, USA), and PKC modulators (PMA, PDBu, Go6983, bisindolylmaleimide I) were provided by Calbiochem (San Diego, CA, USA). The active form of purified PKC-βII was provided by Echelon Biosciences, Salt Lake City, UT, USA. The PCR reaction kit (TOYOBO blend tag) was purchased from Toyobo (Osaka, Japan).

### Site-directed mutagenesis and plasmids

The cDNAs of Stim1 (NM_003156.4) and PKC-βII (NM_001316672) were amplified by PCR using gene-specific primers and cloned into myc-tagged or FLAG-tagged pCS4 expression vectors. Site-directed mutagenesis of the phosphorylation sites was performed by the overlap extension PCR protocol (Ho et al. 1989) with the Quickstart PCR kit (Stratagene) and the primers listed in Table 1. The first round of PCR was performed with primers (1) and (4) or (2) and (3) using Stim1 cDNA in pCS4 + FLAG, followed by the second round of PCR with the PCR products from the first round and the primers (1) and (2) under the following conditions: step 1, pre-denaturation at 94 ℃ for 2 min once; step 2, denaturation at 94 °C for 30 s for 35 cycles; step 3, annealing at 60 ℃ for 30 s; and step 4, extension at 72 ℃ for 2 min. The PCR product was purified using the Qiagen PCR purification kit and cloned into pcDNA5-FRT/TO. The mutation of Ser660 to Ala was confirmed by DNA sequencing (Macrogen, Seoul, Korea). The PKC plasmids, which express wild type PKC-β and the constitutively active truncated (CAT) forms of PKC-α, -β, -δ, -ε, were provided by Dr. Soh (2007). Stim1 cDNAs, including the cytoplasmic region and the three C-terminal sub-regions (Stim1-C1, -C2 and -C3), were cloned into pGEX-4 T-1 plasmids to express the GST-tagged cytoplasmic or the three C-terminal regions of Stim1 as previously described (Jeon et al. 2018). The primers, which were used for PCR amplification and cloning into pcDNA5 FRT TO (Tet-inducible vector), are listed below:

1. Stim1-5’-Hind3-For, 5’- CGC-AAGCTT-GCCATGGATGTATGCGTCCGTCTT -3’,
2. Stim1-3’-XhoI-Rev, 5’- CGC-CTCGAG-**CTA**CTTCTTAAGAGGCTT -3’

**Table 1:** List of primers for Cloning and Sight directed mutagenesis

Gene	Primer name	Directionality	Sequence (5′3’)	Annealing Tm ℃
Stim1	Stim1-T626A & S628A	Forward (For)	CCAGACCCAGACGCACCAGCTCCAGTTGGG	82
		Reverse (Rev)	AACTGGAGCTGGTGCGTCTGGGTCTGGGTC	81
	Stim1-S660A	Forward	GATAATGGCGCTATTGGCGAGGAAACAGAC	73
		Reverse	CTCGCCAATAGCGCCATTATCCTCCTCAGC	76
	Stim1-D76A	Forward	AAACTGATGGCCGATGATGCCAATGGTGAT	74
		Reverse	ATCATCGGCCATCAGTTTGTGGATGTT	71
	Stim1-E87A	Forward	GATGTGGAAGCCAGTGATGAGTTCCTGAGG	74
		Reverse	ATCACTGGCTTCCACATCCACATCACC	73
	Stim1-S575A	Forward	CTGCCTGACGCCCCTGCCCTGGCCAAG	84
		Reverse	CAGGGCAGGGGCGTCAGGCAGTTTCTC	80
	Stim1-S608A	Forward	CCTGGTGGCGCCCCACATTTGGATTCT	78
		Reverse	CAAATGTGGGGCGCCACCAGGTGGGGC	83
	Stim1-S621A	Forward	AGCCCCAGCGCCCCAGACCCAGACACACCA	86
		Reverse	TGGGTCTGGGGCGCTGGGGCTGTGAGAACG	85
PKC	PKC- gamma	Forward	CGCGAATTCAGCTGGTCTGGGCCCCGG	77
		Reverse	CGCCTCGAGTTACATGACGGGCACAGGCA	70
	PKC-beta	Forward	CGCGTCGACGATGGCTGACCCGGCTGC	73
		Reverse	CGCGGATCCTTAGCTCTTGACTTCAGG	54

### Tet-inducible cell lines

The T-REx™ 293 cells were seeded on 10-cm culture dishes in DMEM supplemented with 10% FBS and 1 mg/ml penicillin/streptomycin and incubated until 70% confluent. The cells were then transfected with the Stim1-pcDNA5-FRT/TO and pOG44 vectors at a ratio of 1:9 and with an empty- pcDNA5-FRT/TO vector as control, respectively. Two days later, the cells were selected with 100 µg/ml hygromycin B in normal growth medium. Each selected colony was placed into a separate well on a 24-well culture dish and maintained in DMEM containing 100 µg/ml hygromycin B until confluent. Stim1 protein expression was induced by adding doxycycline at a final concentration of 1 µg/ml into the growth medium and checked by western blotting using rabbit anti-Stim1 antibody (sc-68897, Santa Cruz).

### In vitro kinase assay

The C-terminal region of Stim1 cDNA was divided into 3 sub-regions, which were amplified by PCR with the primers summarized in Table 1 and cloned into GST plasmids (pGEX4T-1). Following the expression, GST control proteins, GST-Stim1 fusion proteins, including GST-Stim1-C-term, -C1, -C2, and -C3, and GST-Stim1-C-terminal mutant proteins, including GST-Stim1-C-term-626A-628A, -660A, and -626A-628A-660A, were purified from *E. coli* as reported before (Jeon et al. 2018). An equal amount (2 µg) of each GST-chimeric protein was mixed with 1 µl of kinase-active PKC-βII in the presence of [γ-^32^P] ATP and kinase buffer (20 mM HEPES pH 7.4, 1 mM DTT, 0.1 mM Na_3_VO_4_, 2 mM EGTA, 20 mM MgCl_2_, and protease inhibitor) for 15 min at 30 ℃. The reaction mixtures were separated on 10% SDS–polyacrylamide gels, transferred onto nylon membranes and autoradiographed by exposure to X-ray film.

### Confocal microscopy

HeLa cells were plated in a 4-well dish (NUNC) at a density of 5 × 10^3^ cells/well and incubated at 37℃, overnight. After incubation, the cells were transfected with Stim1 and/or PKC-β cDNA plasmids as described above, followed by chemical treatment (PMA, TG, Ca^2+^), PBS washings, fixation, permeabilization, and PBS washings as described before Jeon et al. (2018). To detect Stim1 and PKC proteins, we incubated the cells with anti-FLAG and anti-myc antibodies, respectively, for 1 h at 37 ℃, washed them with PBS, and incubated them for 40 min with fluorochrome-conjugated secondary antibodies (Alexa Fluor488- or 594-conjugated antibodies). After mounting, the cells were analyzed using a laser-scanning confocal microscope (Zeiss LSM710). For the activation of ER calcium depletion by ionomycin, HeLa cells were sequentially treated with 100 nM PMA or 10 nM Go6983 for 30 min and 2.5 µM ionomycin for 5 min before imaging with a Nikon-A + confocal microscope at core imaging facility of IBS, Daejeon, Korea.

### Calcium mobilization assay

HeLa and HEK293 cells were incubated with 1 µM Fluo-4 (Invitrogen, USA), a fluorescent Ca^2+^ indicator dye, for 30 min, washed once with Krebs–Ringer-HEPES buffer, and incubated with Krebs–Ringer-HEPES high K^+^ buffer. Then, EGTA (5 mM), TG (2 µM), and Ca^2+^ (2 mM) were added in sequence to the culture medium, and the fluctuation in intracellular Ca^2+^ mobilization was imaged by confocal microscopy (Carl Zeiss LSM710).

## Results

### PKC interacts with Stim1

**Fig.1:**
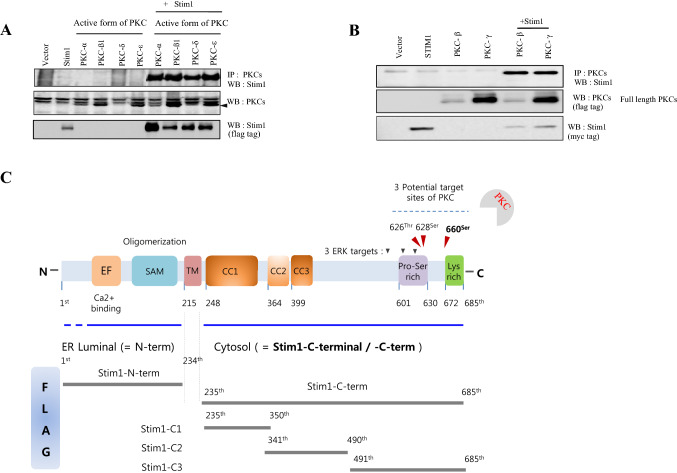
Sstim1 interacts with full length and active forms of PKCs. **A** Stim1 cDNA in pCS4 + (3xmyc-tag) plasmid or constitutive active forms of PKCs in (HA-tagged) pHACE plasmid was transfected alone or together in HEK293 cells. The cells were later lysed in the lysis buffer and the lysates were subjected to centrifugation at 4 °C to remove cell debris from the supernatant. The cleared supernatant was immuno-precipitated with anti-HA antibody and the precipitate was run in the SDS-PAGE gel before western blotting with anti-Stim1 antibody. Both Stim1 and PKC expression in the cell lysates were identified in a separate SDS-PAGE gel run and western blotting using the specific antibodies. eGFP cDNA in pCS2 plasmid was included as a negative control in the experiment. **B** Stim1 cDNA in pCS4 + plasmid (myc tag) and PKC-β or PKC-γ cDNA in in pCS4 + plasmid (flag tag) was transfected in HEK293 cells individually or together. Two days later, the cells were lysed in the cell lysis buffer containing 1% NP 40 and protease cocktail (Roche). **C** The human Stim1 protein was dissected into N-terminal region on the left side, followed by transmembrane (TM) and C-terminal region on the right side in the diagram. The transfected cells were processed as in the above and were immuno-precipitated with Stim1 using the antibody against the flag tag followed by immune blotting with anti myc (PKC-β) antibody before imaging with LAS3000 (Fuji)

For our initial study for PKC-Stim1 interaction, we obtained a set of constitutively active and truncated forms of classical (PKC-α and -β) and novel (PKC-δ and –ε) PKCs from Dr. Soh, Inha Univ., (Kazi and Soh 2007). As shown in Fig. 1A, Stim1 efficiently bound both types of constitutively active PKCs in HEK293 cells. To proceed this study further, we attempted a cloning of the classical type of PKCs using template plasmids from Korea Human Gene Bank and succeed in obtaining a full length of PKC-α and -γ (pCS4 + 3xflag).

Immunoprecipitation of PKC-β or -γ resulted in the co-precipitation of Stim1, confirming the above binding results (Fig. 1B) and subsequently used the PKC-βsince it is one of the most studied PKC in a diverse cellular contexts (Kazi and Soh 2007). To identify the exact region of Stim1 required for the interaction with the PKCs, we divided Stim1 cDNAs into 4 sub-regions as follow; Stim1-N-terminal (ER lumen side), Stim1-C1 (CC1 domain), Stim1-C2 (CC2 and CC3 domain), and Stim1-C3 (C-terminal end domain), and cloned them into pCS4 + FLAG vectors (Fig. 1C). These Stim1 plasmids were co-expressed with active form PKC-beta in HEK293 cells, followed by immunoprecipitation of Stim1 and SDS-PAGE gel running and LC–MS/MS analysis (data not shown).

### PKC phosphorylates the C-terminal region of Stim1

**Fig. 2:**
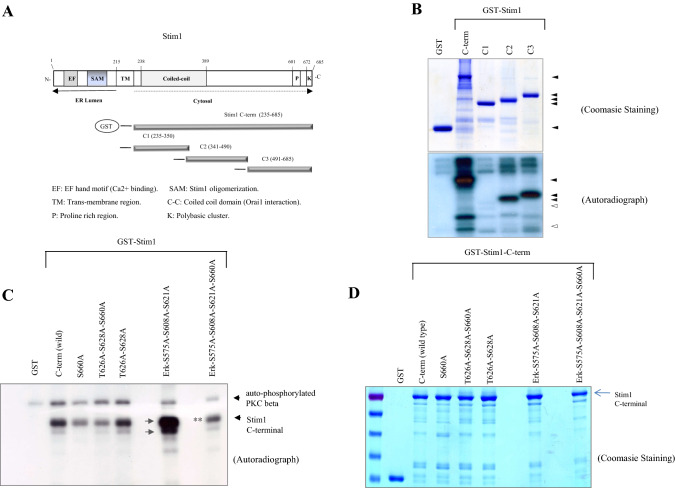
Stim1 phosphorylation by PKC-β. **A** Wild type or deletion forms (C1, C2 and C3) of Stim1 C-terminal cDNAs were cloned in pGEX4T-1 plasmid as in the Schematic diagram with N-terminal GST tag and PKC-β cDNA in pCS4 + plasmid (myc tag) were transfected individually or together in HEK293 cells. The transfected cells were processed as in the above and were immuno-precipitated with Stim1 using the antibody against the flag tag followed by immune blotting with anti myc (PKC-β) antibody before imaging with LAS3000 (Fuji). **B** The GST-Stim1 chimera proteins (GST-Stim1-C-term, -C1, C2 and –C3) were expressed in BL21 bacteria strain with IPTG, followed by a protein purification with Glutathione bead. The purified proteins of GST-Stim1 were mixed with kinase active PKC-b protein in the kinase assay buffer in the presence of radioactive gamma ATP. After the kinase assay, the reaction products were analyzed in the SDS-PAGE gel, and after coommassie staining (the upper box), the dried gel was exposed to X-ray film for radioactive imaging (the lower box). **C**, **D** The GST-Stim1 chimera proteins (GST-Stim1-C-term and 5 point mutant proteins of GST-C-term) were expressed in BL21 bacteria strain with IPTG and purified as in the above before the kinase assay with the purified kinase active PKC and SDS-PAGE gel run and X-ray film imaging. The radioactive SDS-PAGE gel was blotted on the nylon membrane and imaged with X-ray film

Next, we studied whether the classical form of PKC not only interacts with but also phosphorylates the C-terminal region of Stim1 in HEK293 cells. To address this question, in vitro kinase assay was performed with purified GST-Stim1 fusion and a purified PKC- αII (the only one commercially available for PKC-β forms). As shown in Fig. 2B, PKC phosphorylated the C-terminal region of Stim1. The C2 and C3 sub-regions were phosphorylated by PKC, whereas the C1 sub-region and the negative control (GST) were not.

We then attempted to narrow down further the domain of amino acid residues targeted by PKC phosphorylation by liquid chromatography-tandem mass spectrometry (LC–MS/MS). The C-terminal region of Stim1 was (co-)expressed with or without PKC-β in HEK293 cells and treated with either PBS buffer or the DAG analog PMA (Phorbol 12-myristate 13-acetate) (Supplementary data 1). The Stim1 C-terminal region was immuno-precipitated (with or without PKC-β and PMA) and subjected to LC–MS/MS analysis. Although the overall signal level was low, the analysis revealed 10 potential phosphorylation sites, mainly located in the C3 sub-region, as summarized in Fig. S3.

To confirm this result, we conducted site-directed mutagenesis to identify the potential phosphorylation sites by mutating Ser and Thr residues to Ala. Through laborious in vitro kinase assays, we narrowed down the potential target sites to Thr626, Ser628, and Ser660 positions. Finally, Ser660 was identified as the most important phosphorylation site of PKC (Fig. 2C, D). The serine residues at position Ser575, Ser608, and Ser621 in Stim1 are reportedly the phosphorylation sites for ERK and responsible for the increase in SOCE activity (Pozo-Guisado et al. 2010). When all of these 3 Serine residues were mutated to Alanine, the mutant form of Stim1 C-terminal protein was heavily phosphorylated (arrowheads) by PKC. However, mutation of Ser to Ala at 660th residue resulted in a considerable decrease in the overall phosphorylation of Stim1 (marked by 2 asterisks **). This result suggests that ERK and PKC kinases may regulate each other to modulate the Stim1 mediated SOCE. Taken together, our data suggest that PKC phosphorylates multiple residues at the C-terminal region of Stim1 in which Ser660 appears to be one of the major phosphorylation sites.

### PKC inhibits Stim1 puncta formation

**Fig. 3:**
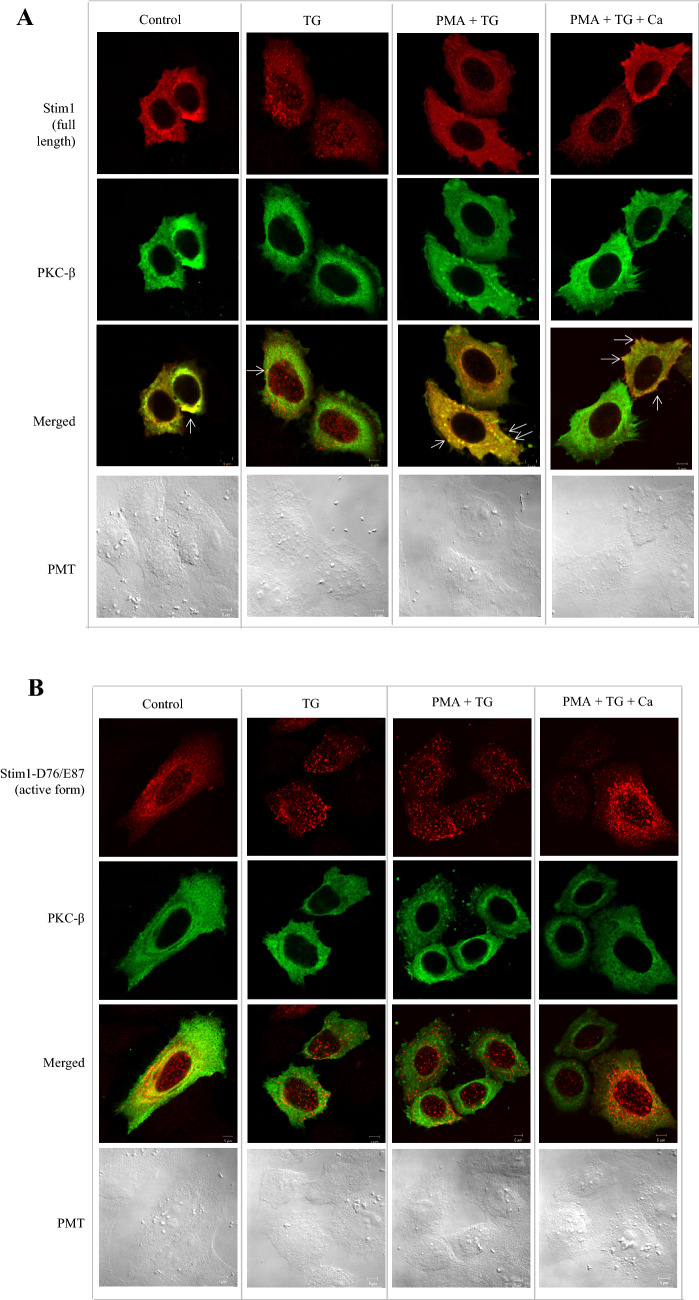
PKC inhibits Stim1 puncta formation by ER store depletion. **A** PKC β (myc-tagged) and Wt- STIM1 (flag tagged) proteins were expressed in HeLa cells overnight before the transfected cells were subjected to confocal imaging process with specific primary monoclonal antibodies and then with the appropriate secondary antibodies with Alexa Fluor 594 for flag tagged Stim1 or Alexa Fluor 488 for myc tagged PKC). The top and the second panels shows the Stim1 and PKC localizations under the 4 different conditions (PBS(control), TG, PMA + TG, PMA + TG + Ca). The third row indicates the merged images of both Stim1 and PKC under 4 different conditions. The bottom row shows bright field images. **B** PKC β (myc-tagged) and Constitutively oligomerizing Stim1 mutant (Stim1-D76/E87) proteins were expressed in HeLa cells and were subsequently processed as in the Fig. 3A

In response to ER Ca^2+^ store depletion, Stim1 undergoes self-oligomerization and forms puncta, which led us to question whether PKC-β has an effect on this process. For this purpose, HeLa cells were transfected with PKC-β- and Stim1-expressing plasmids and treated with phosphate-buffered saline (PBS; control), TG, PMA + TG, or PMA + TG + extracellular Ca^2+^. Confocal imaging showed that TG stimulated Stim1 self-oligomerization (2nd column), whereas PMA, a PKC activator, interfered with this oligomerization process in PMA + TG (3rd column) or PMA + TG + Ca condition (4th column) as Stim1 was diffusely distributed in the cytosol after PMA treatment and co-localized with PKC-β at the cell periphery (marked by open arrows) (Fig. 3A). The Stim1 mutant Asp76Ala/Glu87Ala was reported to self-oligomerize constitutively even in the absence of ER Ca^2+^ store depletion. Thus, we attempted to characterize whether PKC-β interferes with this constitutive self-oligomerization by expressing this mutant in HeLa cells, followed by chemical treatment with TG, PMA + TG, or PMA + TG + extracellular Ca^2+^. As shown in Fig. 3B, the Stim1 mutant appeared as numerous puncta under a basal condition (control, 1st column) or TG condition (2nd column), and PMA treatment did not prevent the mutant form of Stim1 from self-oligomerization either in PMA + TG or PMA + TG + Ca condition.

**Fig. 4:**
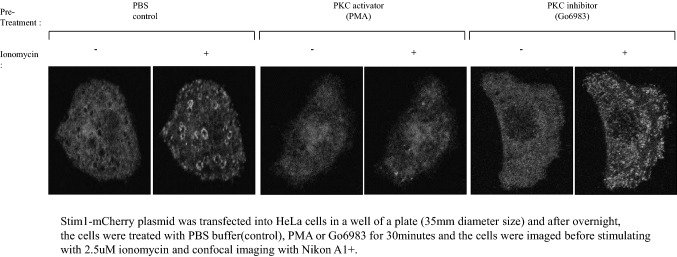
PKC inhibits Stim1 puncta formation by ATP depletion**.** As described in the materials and methods (Confocal microscopy), the HeLa cells were treated with PKC activator-PMA (100 nM final) or PKC ihhibitor-Go6983 (100 nM final), followed by intracellular ATP depletion by 10uM of Oligomycin A. Stim1 localization was analyzed in the bottom region of the cells with anti Stim1 antibody

Besides TG treatment, depletion of the intra-cellular ATP pool by oligomycin A or ionomycin is also known to cause Stim1 puncta formation through ER calcium depletion [Jeon et al. 2018, Morgan and De Bondt 1994]; thus, we investigated the effects of PKC activation (PMA) and inhibition (Go6983) on this process in HeLa cells. Indeed, ionomycin induced puncta formation throughout the cytosol, but PMA treatment abrogated this process, whereas cells treated with the PKC inhibitor Go6983 exhibited numerous puncta in the cytosol (Fig. 4).

### SOCE is regulated by cPKC

**Fig. 5:**
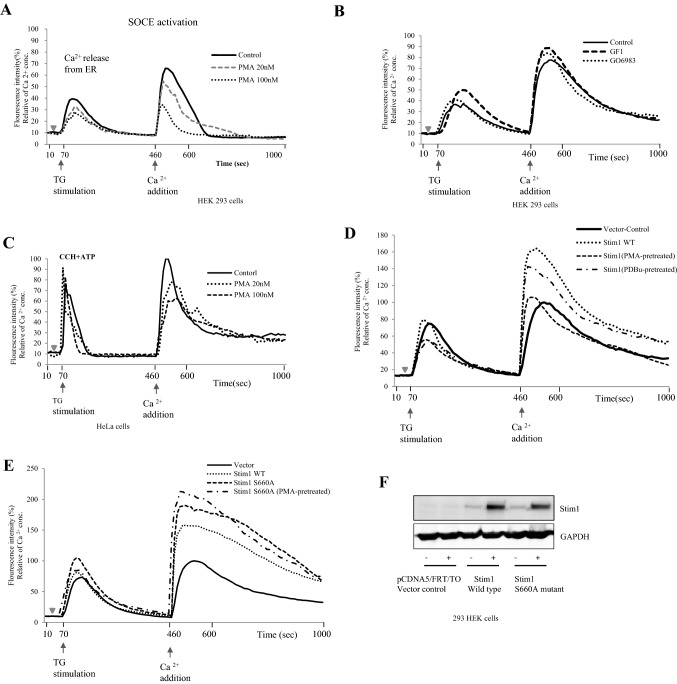
Stim1 mediated SOCE process depends on the PKC-β activity. **A**, **B** Intracellular Calcium concentrations were measured in HEK293 cells pre-loaded with the calcium indicator Fluo-4 and then with PMA/PDBu (PKC activators) or GF1 (PKC inhibitor). To induce ER calcium depletion and then SOCE, the cells were treated with 1 uM thapsigargin (TG) in the absence of extracellular Ca2 and subsequently, extracellular calcium was added to a final concentration of 2 mM to maximize the calcium influx into the activated cells. Intracellular calcium levels are shown as the emission ratio of Fluor4 following excitation at 488 nm. **C** HeLa cells were pretreated with Fluo-4 and then with 2 different concentrations of PKC activator-PMA. ER calcium depletion was induced after addition of 100uM ATP plus 100uM carbachol (ATP + CCh) in calcium-free medium and the cells were processed as in the above. The results are representative of three independent experiments with total 20–25 cells per experiment. **D**, **E** Tet inducible HEK293cell lines, which stably express wild type of Stim1 or mutant form of Stim1-S660A, were pre-loaded with Fluo-4 and then with the PMA. The cells were subsequently were subjected to the same treatment as in the above for SOCE assay. The control cells were transfected with empty Tet inducible plasmid and subjected to the same selection condition compared to the Stim1 expressing stable cell lines

Based on the findings depicted in Figs. 3 and 4, we moved forward to determine whether modulation of PKC activity alters SOCE activity in HEK293 cells. Intracellular Ca^2+^ concentrations were measured after treating cells with the Ca^2+^-sensitive dye Fluo-4 and PKC activators or inhibitors, followed by depletion of ER Ca^2+^ stores with TG and subsequent addition of Ca^2+^. The PKC activators PMA and phorbol 12,13-dibutyrate (PDBu) decreased SOCE activity by approximately 18% and 12%, respectively, compared to that in the DMSO control group. In contrast, the PKC inhibitors Go6983 and bisindolylmaleimide I increased SOCE activity by 14% and 8%, respectively, compared to that in the DMSO control group (Fig. 5A, B). Carbachol is a cholinomimetic drug that activates acetylcholine receptors and intracellular Ca^2+^ mobilization in HeLa cells (Arias-Montano et al. 1994); thus, we tested whether the PKC activator PMA affects the activity of carbachol. PMA down-regulated the SOCE activity, in a dose deponent manner, that is induced by ATP + carbachol by an average of 30% compared with that in the control cells (Fig. 5C). These results indicate that PKC activity is negatively correlated to SOCE activity in HEK293 and HeLa cells.

Next, we established Stim1 stable cell lines for doxycycline-inducible overexpression of the Stim1 wild type and S660A mutant (Fig. 5F). The Stim1 wild type cell line was challenged with PKC activators (PMA and PDBu) and examined for intracellular Ca^2+^ mobilization. Under a basal condition, SOCE activity in this cell line (circular dot line) showed the highest level, which is about 60% more SOCE activity than that of control vector cell line (solid line peak, Fig. 5D). However, treatment with either PMA (dot line) or PDBu (interrupted line) reduced the level of SOCE activity down to that of the vector control cell line.

The cells expressing the S660A mutant exhibited a 1.8 and 1.2 times higher basal level of SOCE than those of the vector control and Stim1 WT respectively, and a treatment with PMA failed to down-regulate SOCE activity. This finding confirmed that residue Ser660 in Stim1 is an important target for PKC phosphorylation and SOCE regulation (Fig. 5E).

## Discussion

Aberrant calcium dynamics has been implicated in a variety of pathological processes, including tumorigenesis, metastasis, and immune deficiency. There is sufficient evidence that alteration of Stim1 and/or Orai1 expression promotes pathogenesis (Karacicek et al. 2019; Picard et al. 2009; Vashisht et al. 2015; Wang et al. 2015]. Besides the protein kinases mentioned above, proline-rich protein tyrosine kinase 2 also increases both SOCE activity through Stim1 phosphorylation and vascular permeability (Yazbeck et al. 2017). Therefore, it seems that the post-translational modifications of Stim1 and Orai1 play a role in (cancer) cell migration and polarized plasma membrane organization, as demonstrated by several groups through RNA interference, gene-knock-out, or Stim1 overexpression systems (Chen et al. 2011; Mo and Yang 2018; Prevarskaya et al. 2014; Tsai et al. 2015; Yang et al. 2009).

Compared to other protein kinases that reportedly increase SOCE through phosphorylation of Stim1, the classical form of PKC appears to be unique since it can bind Ca^2+^ ions, the level of which is transiently increased in the cytosol, and then move to the PM where it interacts with DAG/PMA. The role of activated PKC in the control of Ca^2+^ homeostasis was poorly understood until two groups reported that PKC phosphorylates the N-terminal end of Orai1 to inhibit SOCE (Hooper et al. 2015; Kawasaki et al., 2010).

In this study, we aimed to investigate whether PKC-β, a downstream target of intracellular Ca^2+^, can regulate SOCE through direct interaction and phosphorylation of Stim1 by biochemical assays using two human cell lines—HEK293 and HeLa. Our data demonstrated that PKC interacted with and phosphorylated the C-terminal region of Stim1. A subsequent mapping study revealed that residue Ser660 in Stim1 might be an important target site for PKC phosphorylation. The C-terminal end of Stim1 includes a polybasic, lysine-rich stretch that is proposed to be implicated in plasma/ER membrane tethering of Stim1 (Lacruz and Feske 2015; Lunz et al. 2019). We reason that phosphorylation of Stim1 at Ser660 creates a negative charge that may have an effect on this Stim1-mediated tethering process. The C2-region appears to be phosphorylated in the kinase assay using the GST-Stim1 sub-domain C2 (Fig. 2B), but we could not find any phosphorylation in the LC–MS/MS analysis of the cytosol region of Stim1 (data not shown). We reason that this C2 sub-domain was accessible for PKC phosphorylation when it is expressed in E.coli as a GST-Stim1 C2 chimeric protein, while the same region was inaccessible for PKC when the whole cytosol region was expressed in HEK 293 cells. In support of this idea, the sub-domains of Stim1 are known to undergo a conformation changes and reciprocal interaction between the subdomains (Soboloff et al. 2012).

Chemical activators (PMA, PDBu) and inhibitors (bisindolylmaleimide I, Go6983) of PKC seem to decrease and increase the extracellular Ca^2+^ influx via SOCE, respectively. The finding that SOCE was not inhibited by PMA in cells expressing Stim1-S660A, compared to that in cells expressing wild type Stim1, indicates the significance of the phosphorylation of Stim1 at Ser660 by PKC-β. Taken together, PKC-β may efficiently regulate SOCE through not only Orai1 phosphorylation but also Stim1 phosphorylation.

Confocal imaging revealed that TG induced Stim1 self-oligomerization (i.e., puncta formation) as reported elsewhere (Luik et al. 2008; Liou et al. 2007; Park et al. 2009), whereas pre-treatment with PMA before TG stimulation inhibited Stim-1 self-oligomerization. However, cells expressing a constitutively active (oligomerizing) Stim1 mutant (Asp76Ala/Glu87Ala) maintained their Stim1-mediated puncta pattern regardless of the presence of PMA and TG. It is well known that ionomycin induces Stim1 oligomerization and translocation to plasma membrane to promote its interaction with Orai1. However, our data show that pre-treatment with a PKC activator (i.e., PMA) inhibits the Stim1 oligomerization process, whereas pre-treatment with a PKC inhibitor (i.e., Go6983) did not interfere with the aggregation process as much as the PKC activator did. Therefore, we conclude that PKC plays a role in Stim1 oligomerization to interfere with SOCE.

Under a physiological condition favoring cell proliferation, for example, cell stimulation by a growth factor such as EGF, the EGFR signaling may activate the ERK to promote the intracellular calcium mobilization through followed by SOCE activation and calcium uptake from extracellular environment. Unless this line of activation signaling is not timely switched off, the cells might be subjected to cellular stress leading to growth arrest or even cell death. Therefore, PKC appears to act as a fine tuning switch in such a way that growth factor signaling can activate ERK in favor of SOCE activation, but subsequent PKC activation may result in slow down of the intracellular calcium mobilization through Stim1 and eventually SOCE and calcium uptake across the plasma membrane.

## Conclusions

In summary, our data demonstrate that PKC interacts with the C-terminal region of Stim1 and phosphorylates it at multiple sites. Among these phosphorylation sites, Ser660 appears to be important for regulating SOCE activity. PKC activity is inversely correlated with Stim1 oligomerization and SOCE activity. Further studies are required to investigate the impact of this relationship on cell migration and cancer metastasis.

## Supplementary Information

Below is the link to the electronic supplementary material.

Supplementary file1 (PPTX 558 kb)
