# Supplementary material for: PKC-β modulates Ca2+ mobilization through Stim1 phosphorylation
Source: Genes Genomics. 2022 Mar 7;44(5):571–82. doi: 10.1007/s13258-022-01230-3 (PMC9042968; doi:10.1007/s13258-022-01230-3)
Supplement: Supplementary file 1 — Supplementary file1 (PPTX 558 kb) [file 13258_2022_1230_MOESM1_ESM.pptx]

## Slide 1
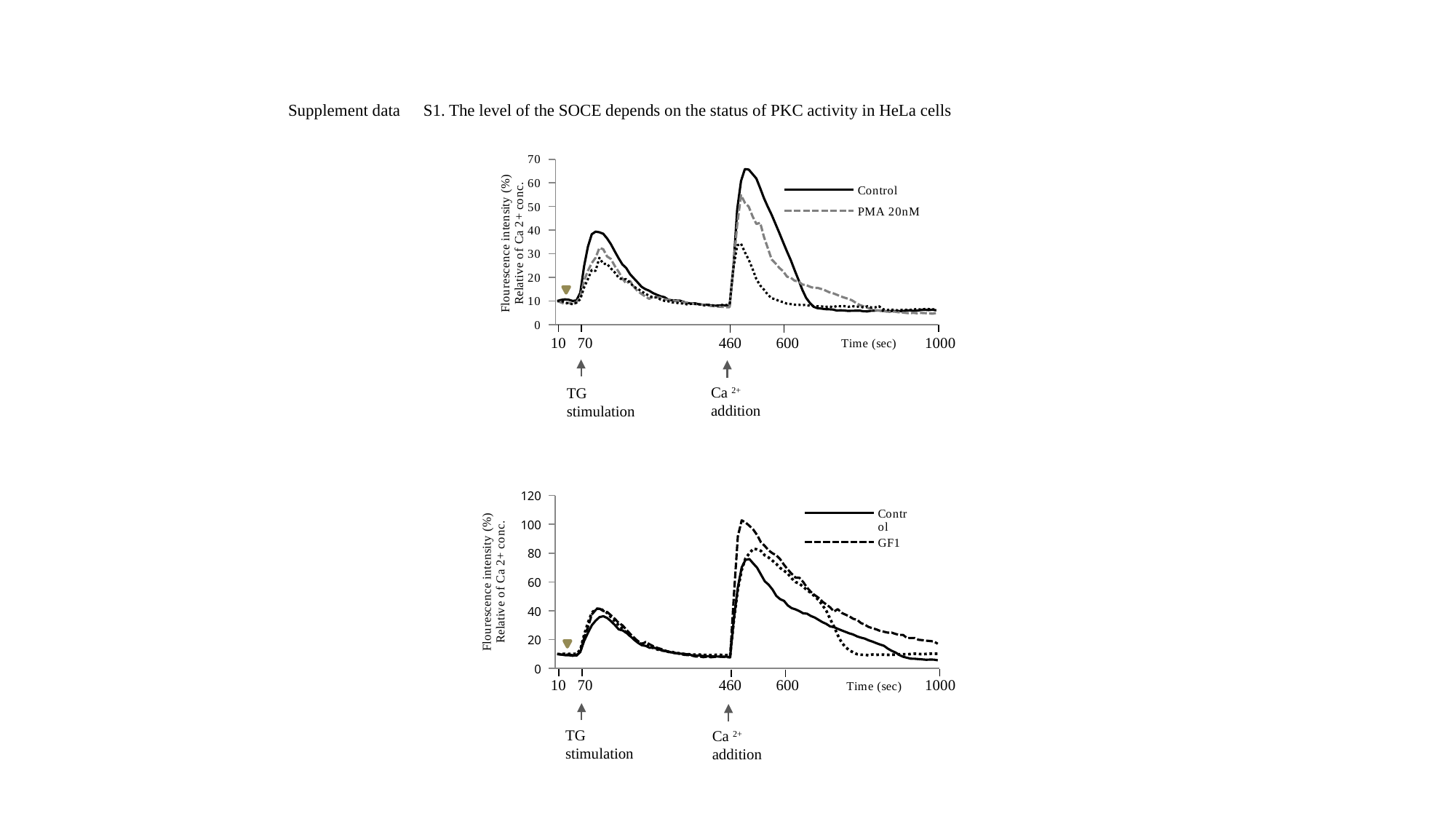

Supplement data
S1. The level of the SOCE depends on the status of PKC activity in HeLa cells
### Chart
| Category | Control | | |
|---|---|---|---|
10 70 460 600 1000
TG
stimulation
Ca 2+
addition
### Chart
| Category | Control | GF1 | GO6983 |
|---|---|---|---|
10 70 460 600 1000
TG
stimulation
Ca 2+
addition

## Slide 2
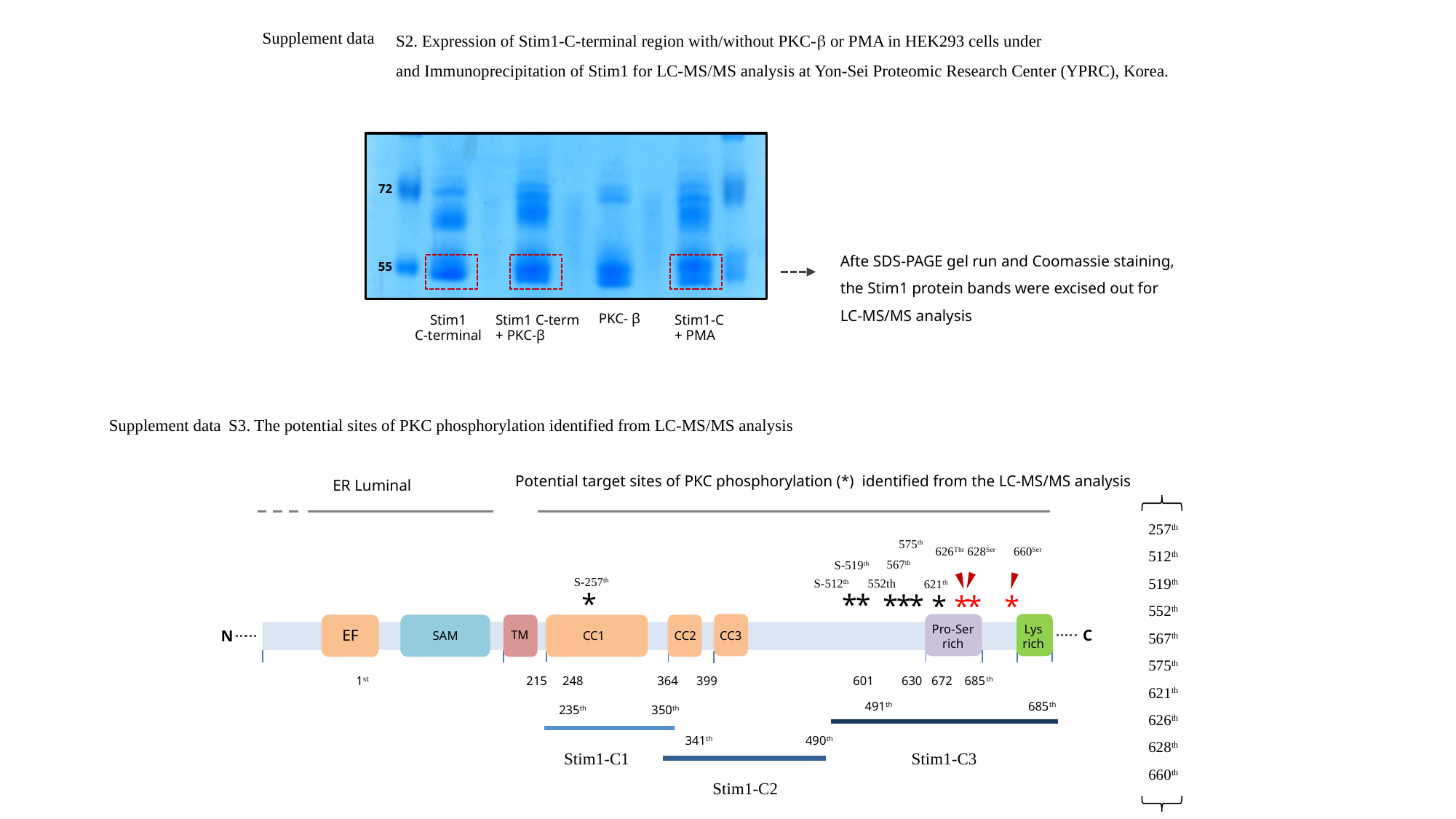

S2. Expression of Stim1-C-terminal region with/without PKC-b or PMA in HEK293 cells under
and Immunoprecipitation of Stim1 for LC-MS/MS analysis at Yon-Sei Proteomic Research Center (YPRC), Korea.
Supplement data
72
55
PKC- β
Stim1
C-terminal
Stim1 C-term
+ PKC-β
Stim1-C
+ PMA
Afte SDS-PAGE gel run and Coomassie staining,
the Stim1 protein bands were excised out for
LC-MS/MS analysis
Supplement data
S3. The potential sites of PKC phosphorylation identified from LC-MS/MS analysis
Potential target sites of PKC phosphorylation (*) identified from the LC-MS/MS analysis
ER Luminal
257th
512th
519th
552th
567th
575th
621th
626th
628th
660th
575th
626Thr 628Ser
660Ser
567th
S-519th
S-257th
552th
S-512th
621th
*
*
*
*
*
*
*
*
*
*
Pro-Ser
rich
Lys
rich
C
EF
N
TM
CC2
CC3
SAM
CC1
1st 215 248 364 399 601 630 672 685th
491th 685th
235th 350th
341th 490th
Stim1-C1
Stim1-C3
Stim1-C2
